# Use of regenerative agents in hand surgery

**DOI:** 10.1186/1753-6561-9-S3-A103

**Published:** 2015-05-19

**Authors:** Sharifah Roohi Ahmad

**Affiliations:** 1 Department of Orthopaedics, Universiti Putra Malaysia, Serdang, 43300, Malaysia

## Introduction

ReGeneraTing Agents (RGTAs) are a family of polymers bioengineered to stabilise heparin-binding growth factors by mimicking HeparanSulphate (HS) thereby protecting them and promoting tissue repair and regeneration. In inflammation, destruction of HS exposes the ExtraCellularMatrix – ECM (structural & cellular proteins within) to the actions of proteases and glycanases which break them down and also act on cytokines and growth factors to prevent adequate repair. In injured tissue, RGTAs would replace destroyed HS by binding to the structural proteins and reconstruct the ECM scaffold. Growth factors will also bind to RGTA and resume position and organization resembling that of non-injured tissue. Hence RGTAs showed they induce a regeneration process by restoring -the proper cellular micro-environment. More recently a RGTA named CACIPLIQ20 was adapted to skin lesions and has shown efficacy in various trials of non-healing leg ulcers.

## Materials and methods

In this pilot prospective study, we applied the same RGTA with meticulous wound care techniques practiced on 10 patients with wounds of varying sizes (average of 13cm²) and depth but with the same underlying theme of poor vascularity and using the recommended sterile application technique twice a week until the wounds healed or over-granulation occurred.

## Results

All wounds eventually healed, even chronic ones. We found that granulation tissue grew again where there was dead skin (Fig below) and no visible underlying blood supply which in usual circumstances would have resulted in loss of limb length,dry gangrene or at best healing by severe scarring. Exposed tendons were also covered with granulation tissue, but instead of a scarred non-mobile digit, simultaneous therapy resulted in a fair range of motion. Full thickness palmar and dorsal wounds also granulated and the dorsal wound healed beautifully reproducing a flexible movable dorsal surface not seen in granulating, scarred healing.

**Figure 1:**
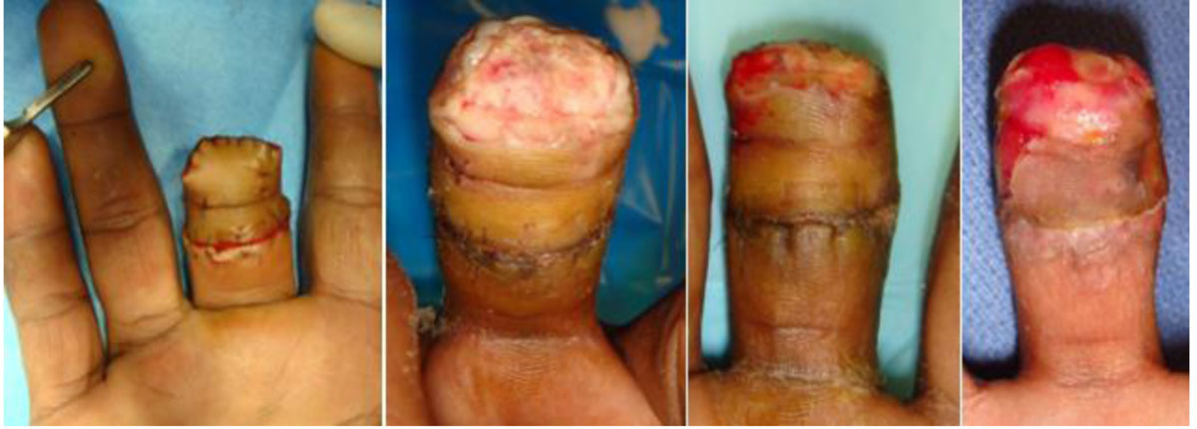


## Conclusion

The revascularisation, development of non-adherent coverage reproducing normal skin features and mobility preserved overlying tendons all suggest that RGTA is a promising alternative treatment.

